# High glucose‐induced complement component 3 up‐regulation via RAGE‐p38MAPK‐NF‐κB signalling in astrocytes: In vivo and in vitro studies

**DOI:** 10.1111/jcmm.13884

**Published:** 2018-09-24

**Authors:** Yuxing Zhao, Cheng Luo, Jinliang Chen, Yue Sun, Die Pu, Ankang Lv, Shiyu Zhu, Jing Wu, Meili Wang, Jing Zhou, Zhiyin Liao, Kexiang Zhao, Qian Xiao

**Affiliations:** ^1^ Department of Geriatrics The First Affiliated Hospital Chongqing Medical University Chongqing China; ^2^ The First People's Hospital of Zunyi Zunyi China

**Keywords:** astrocyte, complement component 3, diabetes, NF‐κB, p38MAPK, receptor for advanced glycation end products

## Abstract

Diabetes is considered as a risk for cognitive decline, which is characterized by neurodegenerative alteration and innate immunity activation. Recently, complement 3 (C3), the critical central component of complement system, has been reported to play a key role in neurodegenerative alterations under pathological condition. Receptor for advanced glycation end products (RAGE) activation is confirmed to mediate several inflammatory cytokines production. However, whether C3 activation participates in the diabetic neuropathology and whether this process is regulated by RAGE activation remains unknown. The present study aimed to investigate the role of C3 in streptozotocin‐induced diabetic mice and high glucose‐induced primary astrocytes and the underlying modulatory mechanisms. The decreased synaptophysin density and increased C3 deposition at synapses were observed in the diabetic brain compared to the control brain. Furthermore, the elevated C3 was co‐localized with GFAP‐positive astrocytes in the diabetic brain slice in vivo and high glucose‐induced astrocytes culture in vitro. Diabetes/high glucose‐induced up‐regulation of C3 expression at gene, protein and secretion levels, which were attenuated by pre‐treatment with RAGE, p38MAPK and NF‐κB inhibitors separately. These results demonstrate that high glucose induces C3 up‐regulation via RAGE‐ p38MAPK‐NF‐κB signalling in vivo and in vitro, which might be associated with synaptic protein loss.

**Abbreviations**
C3: complement component 3
RAGE: receptor for advanced glycation end products
DM: diabetes mellitus
AGEs: advanced glycation end products
STZ: streptozotocin
GFAP: glial fibrillary acidic protein
SYP: synaptophysin
HG: high glucose
NG: normal glucose

## 1 INTRODUCTION

Epidemiologic studies suggest that diabetes mellitus (DM), a complex metabolic disorder, is a risk factor for cognitive decline.^1^, ^2^, ^3^, ^4^ Long‐term diabetes‐induced cognitive decline is characterized by neuropathological alterations including synapse loss, neuronal apoptosis, tau phosphorylation and advanced glycation end products (AGEs).^5^, ^6^, ^7^, ^8^ Recently, there has been an increasing interest in the notion that activated innate immunity is the critical pathogeneses of diabetes‐related cognitive disorder.^3^, ^9^, ^10^ However, the detailed mechanisms of innate immunity activation in diabetic CNS are still ill‐understood.

Complement is the critical part of the innate immunity system that identifies pathogens and damaged cells, which initiates the phagocytosis and proinflammatory responses. Elevated complement proteins in circulation of patients with diabetes have been considered as strong predictors of the development of diabetes and related complications.^11^ Although brain was traditionally considered as an immune‐privileged organ, it has been recently recognized that neuronal cells in the CNS could synthesize many complement system components, which maintain immunosurveillance and biological activities in the brain.^12^, ^13^ Specifically, complement component 3 (C3), the critical central component of three complement cascades,^14^ not only mediates the synaptic pruning and synaptic refinement during brain development,^15^ but also exerts detrimental effects on the CNS in the adulthood.^16^, ^17^, ^18^, ^19^ In the aging mice, C3 overexpression played a causative role in neuron loss and cognitive dysfunction.^20^ In the mice model of Alzheimer's disease, elevated localization of C3 onto hippocampal synapses resulted in synapse loss caused by amyloid‐β.^18^ In addition, the neuronal degeneration mediated by C3 activation was described in the rats injured by intra‐hippocampus injection with kainic acid.^17^ However, whether C3 up‐regulation occurs in the diabetic mice brain and the underlying molecular mechanisms remain unknown. Aberrant C3 activation occurs in inflammatory and oxidative stress condition and participates in the progression of normal aging or neurodegenerative diseases.^21^, ^22^, ^23^, ^24^, ^25^ As the most abundant cells in the brain, astrocytes have been considered as one of the major source of C3 under the diseased condition.^22^, ^26^ Thus, we suggested that C3 production might be up‐regulated in the astrocytes induced by diabetes or high glucose(HG).

Receptor for advanced glycation end products (RAGE), a transmembrane receptor of the immunoglobulin super family, is implicated in the progression of various neurodegenerative diseases by identifying its ligand including AGEs and AGE modified amyloid‐β or tau protein.^27^, ^28^ RAGE can be expressed on the surface of astrocytes and up‐regulation of RAGE has been reported to mediate neuropathology and neuroinflammation.^29^, ^30^ As the downstream effectors of RAGE signalling, p38MAPK and NF‐κB activation play crucial roles in several inflammatory components production.^31^, ^32^, ^33^ However, little is known about the potential involvement of RAGE signalling activation in regulating of C3 production. In the present study, we aimed to investigate whether and how RAGE signalling activation is capable of triggering C3 up‐regulation in the streptozotocin (STZ)‐induced diabetic mice in vivo and HG‐induced primary mouse astrocytes in vitro. Our results indicate that C3 expression increased in the HG‐induced astrocytes as well as in the diabetic mice brain via RAGE‐p38MAPK‐NF‐κB signalling, which could be a potential target to prevent diabetes associated neurodegenerative pathology.

## 2 MATERIALS AND METHODS

### 2.1 Animals and experimental design

Eight‐week‐old male C57BL/6J mice (n = 45, weight 25‐30 g) were obtained from the Animal Center of Chongqing Medical University. Mice were housed in a specific pathogen‐free mouse facility on a 12‐hour light‐dark cycle, with ad libitum access to food and water. Mice were randomly divided into three groups (n = 15 per group): Control group, DM group and DM+ FPS‐ZM1 group. Diabetic mice were induced as previously reported with some modifications,^34^ after a 12‐hour fast, mice received a single 150 mg/kg intraperitoneal injection of STZ (Sigma, St. Louis, MO, USA). Control mice received an equivalent volume of 0.9% saline injection. Fasting blood glucose was measured 3, 7, 10 and 20 days after STZ injection using ACCU‐CHEK Test Strips (Roche Ltd). Mice with fasting blood glucose above 300 mg/dL were considered as diabetic model. FPS‐ZM1 which can cross the blood‐brain barrier is the high‐affinity RAGE‐specific blocker. As previously described with some modifications, 3 months after diabetes induction, mice in the DM+FPS‐ZM1 group received 1 mg/kg/d intraperitoneal injection of FPS‐ZM1 (0.1 mg/mL) (Cayman Chemical, USA) and mice in other groups received an equivalent volume of 0.9% saline injection for 4 weeks.^34^, ^35^, ^36^, ^37^ Then all mice were killed.

### 2.2 Cell culture and treatment

For primary astrocytes culture, astrocytes were prepared from the brains of 1‐to 2‐day‐old C57BL/6J mice pups as previously described with modifications.^38^ Briefly, after removal of meninges, cortices were minced, dissociated in Dulbecco's modified Eagle's (DMEM) medium containing 0.02% papain and were seeded into poly‐D‐lysine‐coated 25T tissue culture flasks (3×10^6^ cells/flask) in DMEM medium (Gibco, USA) containing 25 m mol L^−1^ D‐glucose, 50 U/mL penicillin, 50 mg/mL streptomycin and 10% foetal bovine serum (Gibco, USA). The cultures were maintained at 37°C with 5% CO_2_ and 95% air. Culture medium was changed every other day. After 10 days, cultures were put on an orbital shaker to remove the microglia. Five days after replating, cultures consisted of at least 95% astrocytes as determined by glial fibrillary acidic protein (GFAP) immunofluorescence staining. Astrocytes at approximately 80% confluency were used in this study and incubated respectively, in a serum‐free DMEM containing basal 25 m mol L^−1^ glucose (normal glucose, NG), and serum‐free DMEM with an extra 15 m mol L^−1^ (HG15 m mol L^−1^, HG15 m mol L^−1^) and 30 m mol L^−1^ glucose (HG30 m mol L^−1^) for 24 hours. To rule out the effect of the osmotic stress, the NG culture was added with 30 m mol L^−1^ mannitol and HG15 m mol L^−1^ culture was added with 15 m mol L^−1^ mannitol. To determine the role of related kinases in the C3 production, specific inhibitors for RAGE (FPS‐ZM1, 25 n mol L^−1^), p38MAPK (SB203580, 10 μ mol L^−1^), NF‐κB (PDTC, 100 μ mol L^−1^) were correspondingly added in the cultures for 1 hour prior to the HG incubation. Then, the astrocytes were harvested immediately for the biochemical analyses.

### 2.3 Quantitative RT‐PCR

Total RNA was extracted from hippocampal tissues or from primary mouse astrocytes with TRIzol reagent (Takara, Beijing, China) according to the manufacturer's protocol. The concentration and purity of RNA were measured by a Bio‐Rad SmartSpec Plus (Bio‐Rad, Hercules, CA, USA). Reverse transcription was performed by using All‐in‐One cDNA Synthesis SuperMix (Biomake, TX, USA) according to the manufacturer's instruction. Quantitative RT‐PCR was performed with qPCR SYBR Green PCR Master Mix (Takara, Beijing, China). The primer sequences were as follows: C3 forward, 5′‐AAG CAT CAA CAC ACC CAA CA‐3′; C3 reverse, 5′‐CTT GAG CTC CAT TCG TGA CA‐3′; GAPDH forward, 5′‐AAT GTG TCC GTC GTG GAT CTG A‐3′; GAPDH reverse and 5′‐GAT GCC TGC TTC ACC ACC TTC T‐3′; Data were analysed by the 2^−ΔΔCt^ threshold cycle method and normalized against GAPDH gene.

### 2.4 Western blot analysis

Hippocampal tissues or primary astrocytes were homogenized and lysed in RIPA lysis buffer (Biosky Biotechnology Corporation, Nanjing, China) with a freshly added protease inhibitor cocktail (Roche Diagnostics, Indianapolis, IN, USA), and centrifuged at 14 000 *g* for 15 minutes at 4°C. Supernatant was harvested for western blotting performed as previously described^39^ and equal amounts of protein samples were loaded onto 10% SDS‐PAGE gels, and transferred onto PVDF membranes (Millipore, Merck, Germany). And then membranes were blocked with 5% bovine serum albumin for 1 hour, followed by incubation with specific antibodies: rabbit anti‐ RAGE (1:1000; cat# 16346‐1‐AP;Proteintech, China), rat anti‐C3(1:50;cat# ab11862; Abcam, Cambridge, UK), mouse anti‐ phospho‐p38MAPK (Thr180/Tyr182) (1:1000;cat#5140;Cell Signaling Technology, Danvers, MA), rabbit anti‐p38MAPK (1:1000; cat#8690;Cell Signaling Technology, Danvers, MA, USA), rabbit anti‐ phospho‐NF‐κB(Ser536) and rabbit anti‐ NF‐κB (1:1000;cat#3033; cat# 8242; Cell Signaling Technology, Danvers, MA, USA), and mouse anti‐β‐actin (1:1000; XinBoSheng, Shenzhen, China) overnight at 4°C. Next day, membranes were washed with TBST and incubated with corresponding horseradish peroxidase (HRP)‐conjugated anti‐rabbit IgG and antimouse IgG (1:2000; ZSGB‐BIO, Beijing, China) for 1 hour at temperature. The intensity of the bands obtained was normalized to the β‐actin band, which was determined by using Fusion software (Fujifilm Corp., Tokyo, Japan).

### 2.5 ELISA

Supernatant of primary mouse astrocytes culture was collected and was filtered using a 0.22 μm syringe filter to remove the cellular debris. C3 secretory protein level was determined by using Mouse Complement C3 ELISA Kit (cat#ab157711, Abcam) according to the manufacturer's instructions.

### 2.6 Immunofluorescence

Mice were anaesthetized by 3% pentobarbital sodium (50 mg/kg) and then transcardially perfused with 4% PFA, and the brains were post‐fixed in 4% PFA overnight at 4°C, followed by cryoprotection in 25% sucrose for 12‐24 hours. Tissues were embedded in OCT compound and sagittal section (20 μm thick) were mounted on glass slides and stored at −20°C. Astrocytes fixed by 4% PFA or sections were permeabilized with 0.2% Triton X‐100. After blocking with 10% goat serum in PBS, cells were incubated with primary antibodies: rabbit anti‐GFAP (1:200, catalog #ab7260, Abcam, MA, USA), rat anti‐C3(1:50; cat# ab11862; Abcam, MA, USA)overnight at 4°C. The tissue slides were incubated with mouse anti‐GFAP (1:200, catalog # ab49874, Abcam, MA, USA)rabbit anti‐RAGE (1:300, catalog #ab216329, Abcam, MA, USA), rabbit anti‐GFAP (1:200, catalog #ab7260, Abcam, MA, USA), rat anti‐C3(1:50; cat# ab11862; Abcam, MA, USA) and rabbit anti‐synaptophysin (SYP) (1:200; cat# ab3294) overnight at 4°C. After primary antibody incubation, samples were then rinsed three times for 5 minutes each in PBS and incubated in the appropriate fluorescent‐conjugated secondary antibody (goat antimouse/rabbit IgG 1:200, ZSGB‐BIO, Beijing, China) for 1 hour at 37°C. The cells were counterstained by DAPI (Biosky Biotechnology Corporation, Nanjing, China). Images of cells were captured with a fluorescence microscope (Zeiss, Thornwood, NY, USA). Images of tissue sections within the CA1 region of hippocampus were captured with laser confocal microscopy (Nikon AIR, Tokyo, Japan). Four random images were selected in each group and 3‐4 areas of interest were selected in each image. Integrated optical density within the selected areas was analysed by the ImageJ software.

### 2.7 Statistical analysis

Data are presented as mean ± standard deviation (SD). Statistical significance was carried out by unpaired student's *t* test or one‐way analysis of variance (ANOVA) using GraphPad Prism5. *P* < 0.05 is considered to be significant.

## 3 RESULTS

### 3.1 Elevated C3 was associated with synaptic protein loss in the diabetic brain

**Figure 1:**
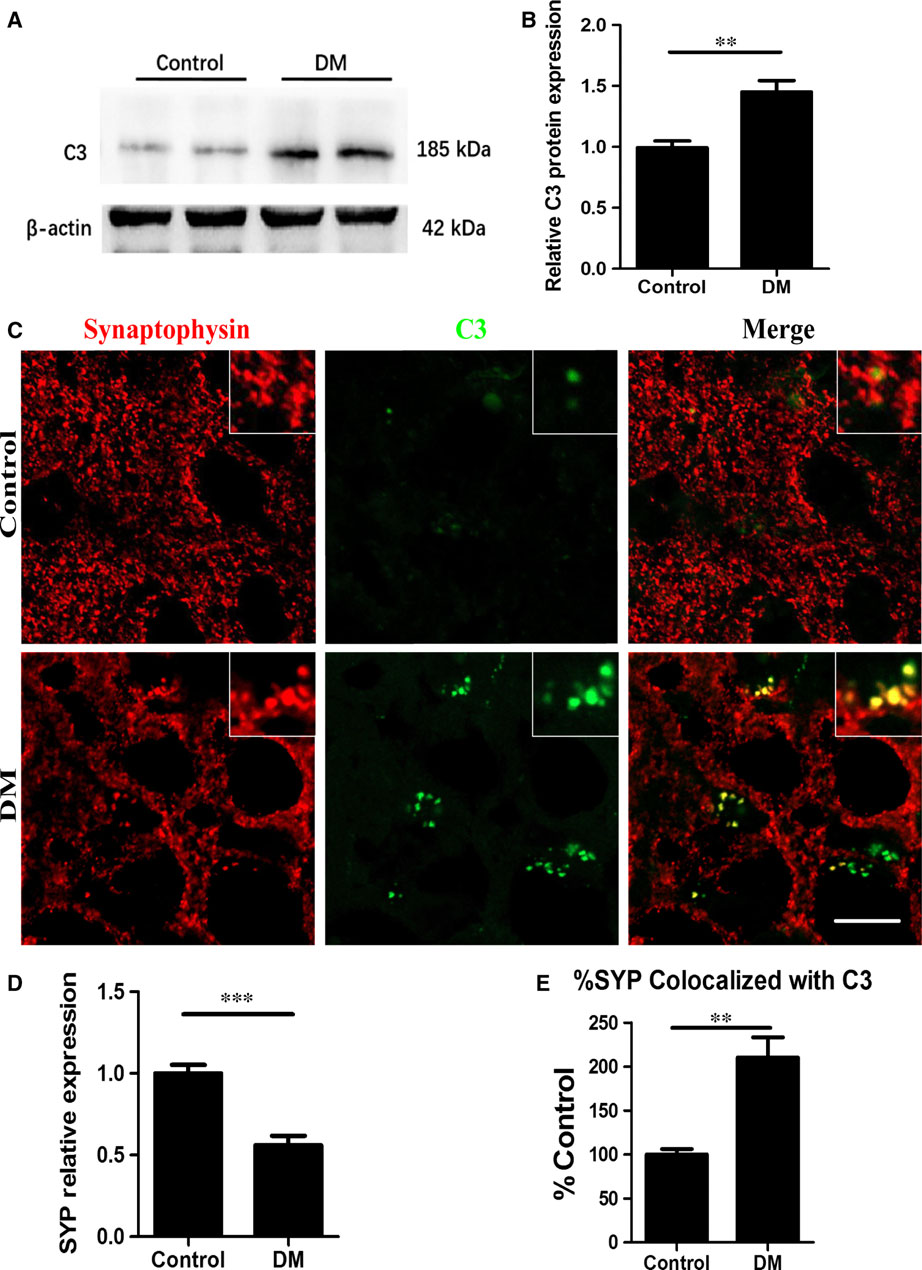
Elevated C3 was associated with synaptic protein loss in the diabetic brain. A, Representative western blots for C3 protein expression in each group; B, Quantification of C3 protein expression by western blots; C, Immunofluorescence staining with synaptophysin (red) and C3 (green) antibodies in the CA1 region of hippocampus. Higher‐magnification inset as indicated. Scale bar = 20 μm; D, Quantification of SYP expression; E, The percentage of SYP co‐localized with C3. C3, complement component 3; DM, diabetes mellitus; SYP, synaptophysin; **P < 0.01; ***P < 0.001; Data are presented as the mean ± SD of three independent experiments (n = 4‐5/group)

Consistent with observations in several neurodegenerative diseases,^22^, ^40^ we found that the C3 protein levels were elevated in the hippocampus of diabetes mice by western blot(DM 1.45 ± 0.09 vs, Con 1 ± 0.56, *P* < 0.01) (Figure 1A, B). To determine whether C3 up‐regulation in the diabetic brain is associated with synaptic degeneration, double‐label immunofluorescence staining with C3 (green) and the presynaptic marker SYP (red) was performed. The decreased SYP density corresponding to increased C3 deposition at synapses were observed in the diabetic brain compared to the control brain, suggesting that elevated C3 deposition might be associated with the synaptic degeneration (DM 0.56 ± 0.06 vs Con 1 ± 0.05, *P* < 0.001; DM 210.33 ± 22.90 vs Con 100 ± 6.24, *P* < 0.01) (Figure 1C‐E).

### 3.2 Up‐regulation of C3 was associated with RAGE signalling activation in the diabetic brain

**Figure 2:**
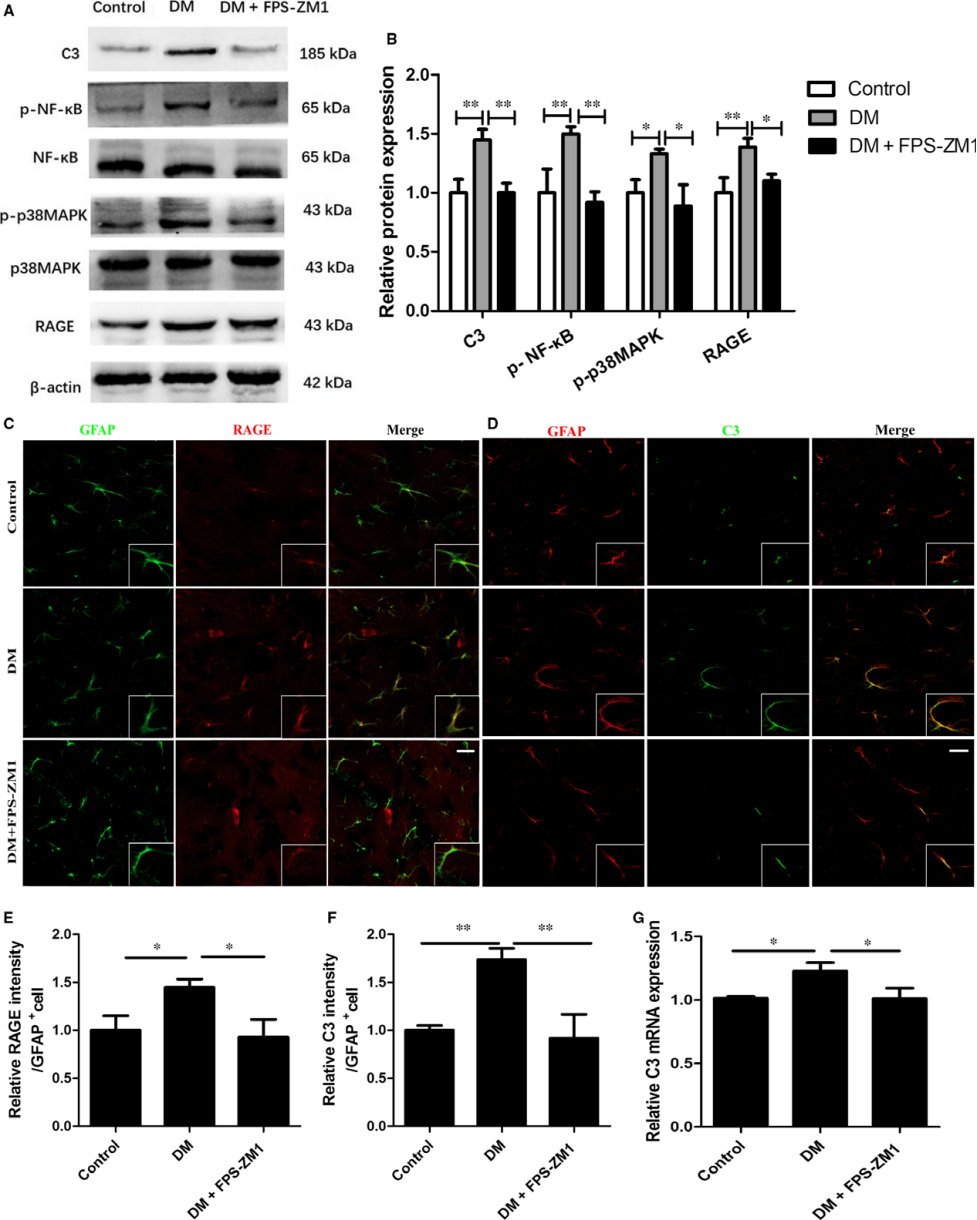
Up‐regulation of C3 was associated with RAGE signalling activation in the diabetic brain. A, Representative western blots for C3, RAGE, p‐NF‐κB, NF‐κB, p‐p38MAPK and p38MAPK protein expressions in hippocampus of each group; B, Quantification of C3, ‐ RAGE, p‐NF‐κB, NF‐κB, p‐p38MAPK and p38MAPK protein expression; C, Immunofluorescence staining with GFAP (green) and RAGE (red) antibodies in the CA1 region of hippocampus. Higher‐magnification inset as indicated. Scale bar = 20 μm; D, Immunofluorescence staining with GFAP (red) and C3 (green) antibodies in the CA1 region of hippocampus. Higher‐magnification inset as indicated. Scale bar = 20 μm; E and F, The average integrated optical density values of RAGE/C3 per GFAP positive cell was quantified; G, Relative expression of C3 mRNA in each group. C3, complement component 3; DM, diabetes mellitus; GFAP, glial fibrillary acidic protein; RAGE, receptor for advanced glycation end products; *P < 0.05; **P < 0.01; Data are presented as the mean ± SD of three independent experiments (n = 4‐5/group)

It has been documented that RAGE activation induced inflammatory response, which impairs the neuronal structure and function. As the critical component of the innate immune system, C3 has been reported to be involved in neuroinflammation and neuropathology.^17^ We utilized the STZ‐induced diabetic mice to determine whether RAGE signalling is associated with C3 production. By western blot, we observed up‐regulated RAGE protein expression in the diabetic mice as compared to the control group (DM 1.39 ± 0.07 vs Con 1 ± 0.13, *P* < 0.01), which were down‐regulated in the diabetic mice treated with FPS‐ZM1 (FPS‐ZM1+DM 1.1 ± 0.06 vs DM 1.39 ± 0.07, *P* < 0.05) (Figure 2A‐B). Meanwhile, an attenuation of C3 protein expression was observed in diabetic mice treated with FPS‐ZM1 as compared to the DM group (FPS‐ZM1+DM 1.01 ± 0.08 vs DM 1.45 ± 0.09, *P* < 0.01) (Figure 2A‐B). In addition, phosphorylation levels of p38MAPK and NF‐κB increased in the DM group as compared to the control group (DM 1.33 ± 0.04 vs Con 1.0 ± 0.11, *P* < 0.05; DM 1.50 ± 0.06 vs Con 1 ± 0.20, *P* < 0.01), which were reversed by treatment with FPS‐ZM1(FPS‐ZM1+DM 0.89 ± 0.18 vs DM 1.33 ± 0.04, *P* < 0.05; FPS‐ZM1+DM 0.92 ± 0.09vs DM 1.50 ± 0.06, *P* < 0.01) (Figure 2A‐B). These results suggest that p38MAPK and NF‐κB kinases are downstream of RAGE activation, which might mediate C3 production in the diabetic brain.

In consistent with western blots, the double‐label immunofluorescence staining in the brain slides showed enhancement of RAGE immunoreactivity (red) in the astrocytes stained by GFAP (green) in the CA1 region of diabetic hippocampus compared to the control group (DM 1.45 ± 0.09 vs Con 1 ± 0.15, *P* < 0.05), which were significantly attenuated by the FPS‐ZM1 treatment (FPS‐ZM1+DM 0.93 ± 0.18 vs DM 1.45 ± 0.09, *P* < 0.05) (Figure 2C, E). Furthermore, co‐localization of C3 and GFAP showed that C3 immunoreactivity (green) increased in the astrocytes of the diabetic brain as compared to the control group (DM 1.74 ± 0.12 vs Con 1 ± 0.05, *P* < 0.01) (Figure 2D, F). However, the FPS‐ZM1 treatment significantly reduced the C3 immunoreactivity in the diabetic brain (FPS‐ZM1+DM 0.92 ± 0.25 vs DM 1.74 ± 0.12, *P* < 0.01) (Figure 2D,F), suggesting that RAGE activation might partly mediate the astrocytic C3 production. Furthermore, C3 mRNA elevated in the DM group as compared to the control group (DM 1.23 ± 0.06 vs Con 1 ± 0.01, *P* < 0.05), which could be ameliorated by the FPS‐ZM1 treatment (FPS‐ZM1+DM 1.01 ± 0.08 vs DM 1.23 ± 0.06, *P* < 0.05) (Figure 2G).

### 3.3 High glucose increased C3 synthesis and secretion in astrocytes in vitro

**Figure 3:**
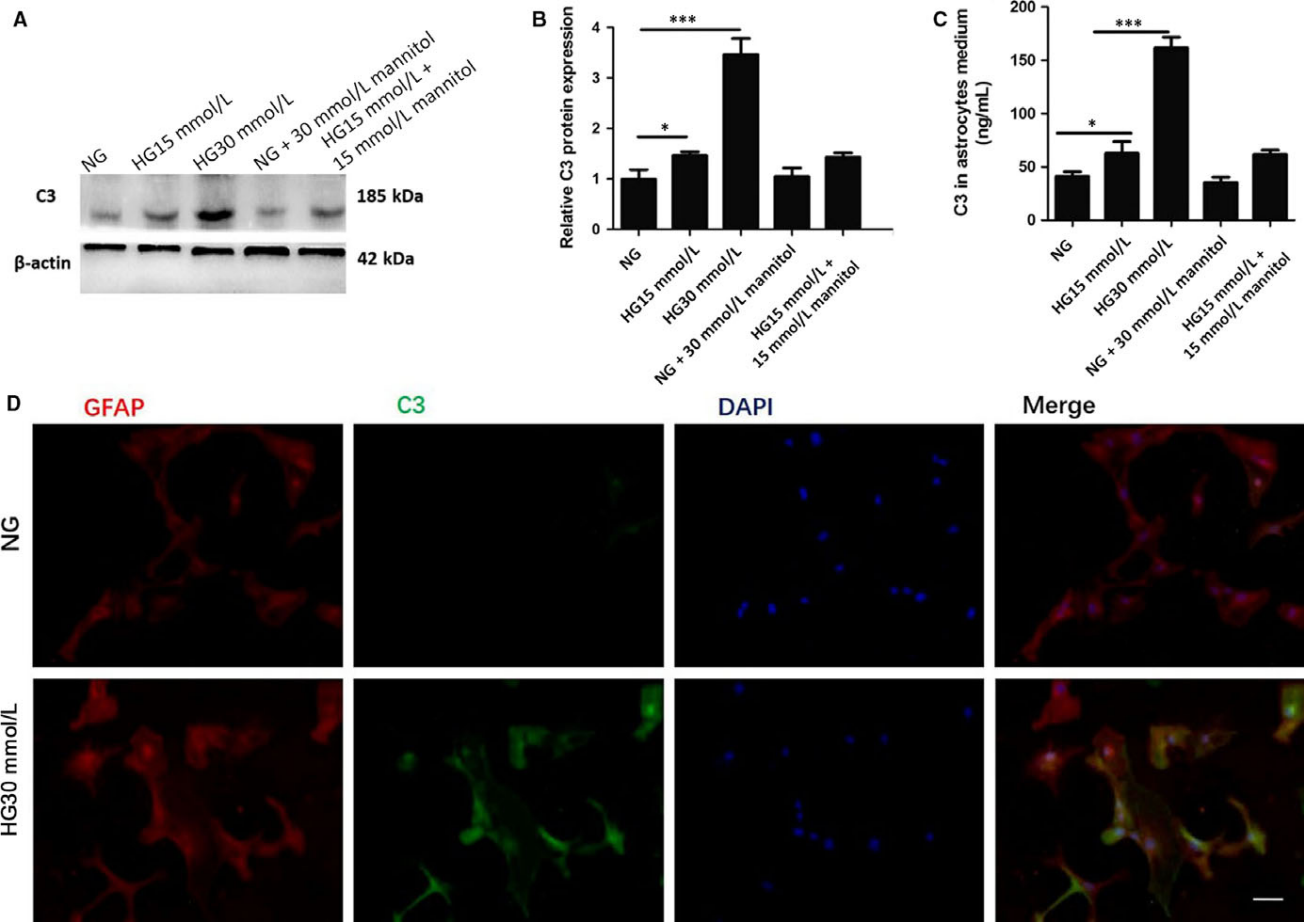
High glucose increased C3 synthesis and secretion in astrocytes in vitro. A, Representative western blots for C3 in astrocytes under different concentration of glucose; B, Quantification of C3 protein expression by western blots; C, Level of C3 secretion in the astrocyte medium measured by ELISA; D, Immunofluorescence staining with GFAP (red) and C3 (green) antibodies and nucleus was counterstained with DAPI (blue). Scale bar = 50 μm; C3, complement component 3; GFAP, glial fibrillary acidic protein; NG, normal glucose; HG, high glucose; *P < 0.05; ***P < 0.001; Data are presented as the mean ± SD of three independent experiments

We established in vitro model to determine the effect of different concentration of glucose (NG, HG15 m mol L^−1^, HG30 m mol L^−1^) on C3 production in astrocytes at protein and secretion levels by western blot and ELISA. Results showed that C3 protein and secretion levels significantly increased in the HG15 m mol L^−1^ and HG30 m mol L^−1^ groups (HG15 m mol L^−1^ 1.46 ± 0.08 vs NG 1 ± 0.19, *P* < 0.05; HG15 m mol L^−1^ 62.67 ± 11.02 vs NG 41.33 ± 4.16, *P* < 0.05; HG30 m mol L^−1^ 3.46 ± 0.32 vs NG 1 ± 0.19, *P* < 0.001; HG30 m mol L^−1^ 161.67 ± 10.12 vs NG 41.33 ± 4.16, *P* < 0.001) (Figure 3A‐C). In addition, to rule out the effect of osmotic stress on C3 production, astrocytes were cultured in the NG group added with 30 m mol L^−1^ mannitol or in HG15 m mol L^−1^ group added with 15 m mol L^−1^ mannitol as high osmolarity control. However, there was no additional effect of mannitol in C3 expression at protein and secretion levels (*P* > 0.05) (Figure 3A‐C), suggesting that C3 expression is not influenced by osmotic stress. As the obvious effect of HG30 m mol L^−1^ on the C3 production, we decided to use the 30 m mol L^−1^ glucose as the condition for the subsequent experiments. Furthermore, to visualize the C3 expression in astrocytes under HG30 m mol L^−1^, we co‐staining of C3 (green) with GFAP (red) antibodies by immunofluorescent assay. Images showed that C3 immunofluorescence obviously increased in the astrocytes incubated in HG30 m mol L^−1^ as compared to the NG group (Figure 3D).

### 3.4 RAGE activation was required for high glucose‐induced C3 up‐regulation in vitro

**Figure 4:**
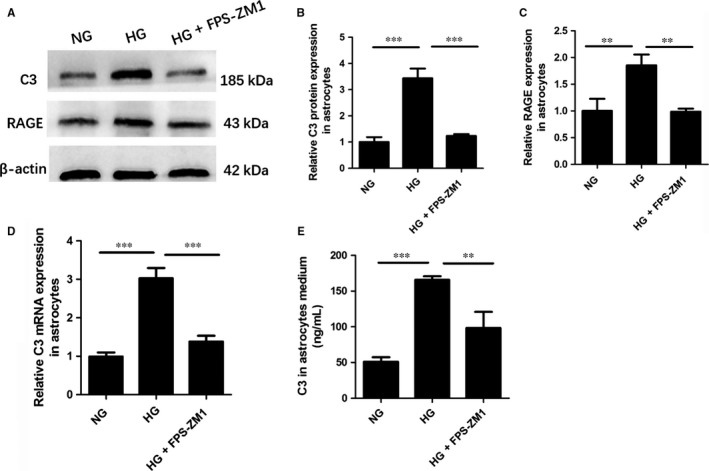
RAGE activation was required for high glucose‐induced C3 up‐regulation in vitro. A, Representative western blots for C3 and RAGE in astrocytes in each group; B, Quantification of C3 protein expression of astrocytes by western blot; C, Quantification of RAGE expression by western blot; D, Relative C3 mRNA expression in primary astrocytes measured by qRT‐PCR; E, Analysis of C3 secretion in astrocyte medium determined by ELISA; C3, complement component 3; RAGE, receptor for advanced glycation end products; NG, normal glucose; HG, high glucose; **P < 0.01; ***P < 0.001; Data are presented as the mean ± SD of three independent experiments

We investigated whether there is a possible relationship between C3 production and RAGE activation in astrocytes under HG condition. We confirmed that HG activated RAGE in astrocytes by western blot. Results showed that RAGE expression elevated in astrocytes incubated in HG medium compared to the astrocytes incubated in NG medium (HG 1.39 ± 0.07 vs NG 1 ± 0.13, *P* < 0.01), which was reduced by pre‐treatment with FPS‐ZM1 (FPS‐ZM1+HG 1.09 ± 0.06 vs HG 1.39 ± 0.07, *P* < 0.01) (Figure 4A, C). Moreover, results from PCR and western blot demonstrated that up‐regulation of C3 mRNA and protein levels in astrocytes under HG condition was attenuated by pre‐treatment with FPS‐ZM1 (FPS‐ZM1+HG 1.38 ± 0.15 vs HG 3.02 ± 0.27, *P* < 0.001; FPS‐ZM1+HG 1.24 ± 0.06 vs HG 3.44 ± 0.37, *P* < 0.001) (Figure 4A, B, D). To examine the involvement of RAGE in the C3 secretion from astrocytes under the HG environment, we used ELISA assay to measure the C3 level in the medium of NG, HG and HG+ FPS‐ZM1 groups. Pre‐treatment with FPS‐ZM1 resulted in a reduction in C3 secretion from astrocytes under HG condition as compared to the astrocytes incubated in HG alone (FPS‐ZM1+HG 98.33 ± 22.54 vs HG 165.67 ± 5.73, *P* < 0.01) (Figure 4E). These findings suggest that RAGE activation in astrocytes induced by HG contributes to an increase in both C3 intracellular and extracellular contents.

### 3.5 P38MAPK phosphorylation was required for RAGE‐mediated C3 up‐regulation in vitro

**Figure 5:**
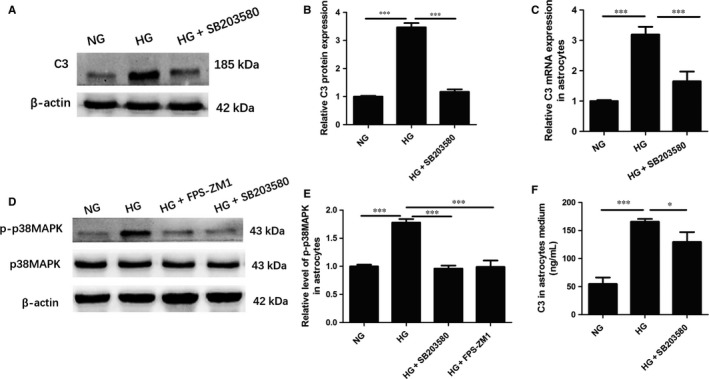
P38MAPK phosphorylation was required for RAGE‐mediated C3 up‐regulation in vitro. A, Representative western blots for C3 in astrocytes in each group; B, Densitometric analysis of C3 protein expression; C, Relative C3 mRNA expression in primary astrocytes; D, Representative western blots for p‐p38MAPK and p38MAPK in astrocytes; E, Quantification of p‐p38MAPK expression; F, Quantification of C3 secretion in astrocyte medium; C3, complement component 3; NG, normal glucose; HG, high glucose; *P < 0.05; ***P < 0.001; Data are presented as the mean ± SD of three independent experiments

It has been documented that p38MAPK is involved in the down‐stream component of RAGE activation signalling and mediates inflammatory response and neuronal injury.^41^, ^42^ In consistent with results in vivo, we observed that FPS‐ZM1 obviously suppressed the activation of p38MAPK in astrocytes under HG incubation (FPS‐ZM1+HG 0.99 ± 0.11 vs HG 1.78 ± 0.06, *P* < 0.001) (Figure 5D, E). Furthermore, to explore the role of p38MAPK activation in the C3 production of HG‐induced astrocytes, we treated the astrocytes with p38MAPK inhibitor SB203580 (10 μ mol L^−1^) for 1 hour prior to the HG incubation. Down‐regulation of p38MAPK phosphorylation was observed in astrocytes of HG+ SB203580 group (SB203580+HG 0.96 ± 0.05 vs HG 1.78 ± 0.06, *P* < 0.001) (Figure 5D, E). In addition, C3 mRNA and protein in cell extracts and C3 secretion level in medium elevated under HG condition, which were abolished by the pre‐treatment with SB203580 (SB203580+HG 1.65 ± 0.32 vs HG 3.19 ± 0.26, *P* < 0.001; SB203580+HG 129.67 ± 17.62 vs HG 3.47 ± 0.15, *P* < 0.001; SB203580+HG 0.96 ± 0.05 vs HG 165.67 ± 5.13, *P* < 0.05) (Figure 5A, B, C, F). These data verified that HG‐induced p38MAPK phosphorylation may participate in RAGE‐mediated elevation of C3 synthesis and secretion.

### 3.6 C3 expression was dependent on the NF‐κB activation induced by high glucose in vitro

**Figure 6:**
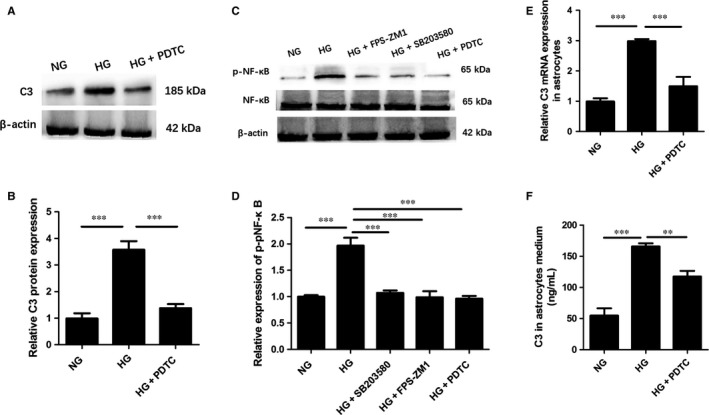
C3 expression was dependent on the NF‐κB activation induced by high glucose in vitro. A, Representative western blots for C3 in astrocytes in each group; B, Quantification of C3 protein expression; C, Representative western blots for p‐NF‐κB in astrocytes in each group; D, Quantification of p‐NF‐κB expression; E, Relative C3 mRNA expression in primary astrocytes; F, Quantification of C3 secretion in astrocyte medium determined by ELISA. C3, complement component 3; GFAP, glial fibrillary acidic protein; NG, normal glucose; HG, high glucose; **P < 0.01; ***P < 0.001; Data are presented as the mean ± SD of three independent experiments

NF‐κB has been reported as the transcription factor that induces C3 expression in various cell types.^40^, ^43^ Consistent with observations in vivo, HG induced the increasing NF‐κB phosphorylation in astrocytes as compared to the NG group (HG 1.97 ± 0.14 vs NG 1.00 ± 0.02, *P* < 0.001) (Figure 6C, D). To determine whether phosphorylated NF‐κB (p‐NF‐κB) is involved in the C3 production in astrocytes treated by HG, we pre‐treated astrocytes with NF‐κB inhibitor PDTC (100 μ mol L^−1^) for 1 hour, followed by the HG incubation. The results from western blot analysis showed that pre‐treatment with PDTC blocked NF‐κB phosphorylation under HG condition (PDTC+HG 0.96 ± 0.05 vs HG 1.97 ± 0.14, *P* < 0.001) (Figure 6C, D). Furthermore, pre‐treatment with PDTC attenuated HG‐induced up‐regulation of C3 protein and mRNA expression, as well as extracellular C3 content, indicating that NF‐κB activation participates in this process (PDTC+HG 1.38 ± 0.16 vs HG 3.58 ± 0.31, *P* < 0.001; PDTC+HG 1.50 ± 0.31 vs HG 2.99 ± 0.07; *P* < 0.001; PDTC+HG 87.33 ± 7.5 vs HG 165.67 ± 5.13, *P* < 0.01) (Figure 6A, B, E, F). To further explore the role of RAGE‐dependent p38MAPK activation in HG‐induced NF‐κB phosphorylation, pre‐treatment of astrocytes with either FPS‐ZM1 or SB203580 markedly decreased the NF‐κB phosphorylation level (FPS‐ZM1+HG 0.99 ± 0.11 vs HG 1.97 ± 0.14, *P* < 0.001; SB203580+HG 1.07 ± 0.05 vs HG 1.97 ± 0.14, *P* < 0.001) (Figure 6C, D), which suggests that HG‐induced NF‐κB activation in astrocytes via RAGE‐p38MAPK. Our data indicate that HG‐induced elevated C3 production might be partly through RAGE‐p38MAPK‐NF‐κB signalling activation in astrocytes.

## 4 DISCUSSION

Diabetes mellitus has been considered as an increased risk for cognitive decline.^2^ Clinical and animal studies have reported that neuroinflammation is one of the major causative factors of diabetes related neurodegenerative pathology.^9^ However, the exact molecular mechanisms are not elucidated enough. In the current study, our results first demonstrate that HG induces C3 activation in astrocytes in vivo and in vitro, which participates in the neuronal degenerative process in the diabetic brain. We also show for the first time the RAGE/p38MAPK/NF‐κB signalling‐dependent mechanism of C3 up‐regulation in astrocytes under diabetic/HG condition.

Complement C3, the pivotal central component of three complement cascades, has been recently suggested to play a key role in the neurodegenerative diseases.^12^, ^19^, ^44^ Astrocytes, which are sensitive to inflammatory stimuli and oxidative insults, has been considered as the primary source of C3.^21^, ^40^ Elevated expression of C3 protein has been implicated in Alzheimer's Disease,^18^, ^40^ Parkinson's disease,^45^ multiple‐system atrophy^23^ and HIV dementia.^46^ Given the potential importance of aberrant C3 activation in neurodegenerative pathology, it is necessary to determine whether C3 activation occurs in the diabetic CNS pathology. Using PCR and western blot, we showed the elevation of C3 gene and protein levels in the diabetic brain in vivo. In vitro study, we observed that HG incubation induced an up‐regulation of intracellular and extracellular C3 levels in a concentration dependent manner. In agreement with the observation from other report,^40^ enhancement in C3 fluorescence was localized in astrocytes of the diabetic brain in vivo as well as under HG condition in vitro. In addition, increasing C3 secretion from astrocytes exposure to HG30 m mol L^−1^ was observed in vitro. Regarding osmotic stress, we found no effect of osmotic stress on the C3 production in astrocytes. These results presented above indicate that HG induces an increase in both C3 synthesis and secretion from astrocytes. Furthermore, as our previous finding reported,^47^ decreased SYP density was found in the DM group as compared to the control group. Importantly, C3 not only plays a crucial role in synapse pruning in the developing mouse visual system,^15^ but also mediates synaptic loss during development of aging^20^ and disease.^16^, ^18^, ^40^ In consistent with their observation, we found that a significant higher percentage of SYP co‐localized with C3 was in the diabetic brain than in the control brain, suggesting that enhanced C3 might mediate the synaptic degeneration in the diabetic brain. However, whether and how C3 activation directly causes synaptic loss has not been elucidated in this study, which need further experiment.

RAGE, which is expressed at a low level under physiologic condition, can be elevated by its ligands under chronic inflammation condition.^48^ RAGE up‐regulation can induce several pathologies, including neuroinflammation, oxidative stress and neuronal degeneration.^29^, ^32^, ^49^, ^50^ RAGE is presented in various cell types in the CNS,^49^, ^51^ including astrocytes. It has been reported that RAGE activation by D‐galactose increased interleukin (IL)‐1β, IL‐6, and tumour necrosis factor‐α (TNF‐α) levels from astrocyte in mice.^52^ Aβ_42_‐induced reactive oxygen species production in primary astrocytes via RAGE activation.^53^ Elevated RAGE in astrocytoma cells activates NF‐κB and promotes the expression of TNF‐α.^50^ Since C3 is the critical component in immune system, which is up‐regulated under the inflammatory condition, we suggested that RAGE activation might potentially regulate C3 production. Therefore, we investigated the possible role of RAGE activation in the mechanism of C3 production in the diabetic brain and astrocytes under HG condition. In the current work, we confirmed the RAGE up‐regulation in astrocytes under diabetic condition in vivo and in vitro. Subsequently, treatment of diabetic mice with FPS‐ZM1, a recently developed the high‐affinity RAGE‐specific inhibitor, can suppress the RAGE expression and phosphorylation of down‐stream protein p38MAPK as well as NF‐κB in diabetic brain tissues. In agreement with previous data from other laboratory reports, FPS‐ZM1 attenuated AGEs‐mediated NF‐κB phosphorylation in rat hippocampus^36^ and RAGE inhibition reduced the p38MAPK activation in Aβ‐induced‐mice model.^32^ In addition, the FPS‐ZM1 treatment ameliorated the up‐regulation of C3 mRNA and protein levels in diabetic hippocampus tissue as well as C3 immunoreactivity in astrocytes of the diabetic brain section. Similarly, pre‐incubation of astrocytes with FPS‐ZM1 under HG incubation decreased the RAGE activation and C3 production at mRNA, protein and secretion levels. Together with our data in vivo and in vitro, RAGE activation associated signalling might at least, in part, be involved in the C3 elevation under HG condition.

As the critical down‐stream effector of RAGE, p38MAPK activation is well known to mediate inflammatory response. In vitro study, p38MAPK activation was observed in astrocytes induced by HG, which was abolished by FPS‐ZM1 pre‐treatment, confirming that HG‐induced p38MAPK phosphorylation in astrocytes is dependent on RAGE activation. Previous studies have reported that up‐regulation of C3 gene expression and secreted levels were regulated by the inflammatory stimuli via activating p38MAPK kinase.^21^, ^43^ In accordance with this statement, we observed that pre‐treatment with SB203580, the p38MAPK inhibitor, significantly suppressed the induction of C3 gene and protein expression in astrocytes by HG incubation, along with decreased C3 release in medium. Therefore, our data indicate that p38MAPK plays a possible role in RAGE‐mediated C3 regulation.

The role of NF‐κB in regulating C3 transcription is broadly discussed in various cell types or tissue.^43^, ^54^ Here, we observed elevated p‐NF‐κB expression in astrocyte of the HG group compared to the NG group. However, there were decreased p‐NF‐κB protein levels in the either FPS‐ZM1+ HG group or SB203580+ HG group as compared to the HG group. These data suggest the involvement of NF‐κB activation in the RAGE signalling. Using NF‐κB pharmacological inhibitor PDTC, we found that HG‐induced p‐NF‐κB was significantly blocked. Moreover, gene, protein and secretion levels of C3 were down‐regulated by pre‐treatment with PDTC, which was in agreement with the previous work showing that the overexpression of the NF‐κB inhibitor I‐κBα in human foetal astrocytes blocked the C3 promoter induction by HIV.^22^ Collectively, RAGE‐mediated p38MAPK/NF‐κB activation might probably be involved in the process of C3 up‐regulation in astrocytes under HG condition.

## 5 CONCLUSION

Our study demonstrates that HG induces C3 up‐regulation in astrocytes in vivo and in vitro, which probably mediates the synaptic degeneration in the diabetic brain. Diabetes‐induced C3 up‐regulation in astrocytes might be dependent on the RAGE activation associated p38MAPK/NF‐κB signalling.

## CONFLICT OF INTEREST

The authors declare that there are no conflicts of interest.

## AUTHORS’ CONTRIBUTIONS

YXZ and QX designed the study and wrote the manuscript. JLC, YS and CL participated in DM model establishment. DP, AKL, SYZ and MLW performed the Western blot, and PCR. JZ and ZYL performed immunofluorescence staining and ELISA. JW and KXZ analysed the data. All authors approved the final manuscript.

## COMPLIANCE WITH ETHICAL STANDARDS

All procedures were performed in approval of Committee on Animal Research of Chongqing Medical University.
